# Supplementary material for: Bird protection treatments reduce bird-window collision risk at low-rise buildings within a Pacific coastal protected area
Source: PeerJ. 2022 Mar 22;10:e13142. doi: 10.7717/peerj.13142 (PMC8953498; doi:10.7717/peerj.13142)
Supplement: Supplemental Information 1 — Each survey included all 11 façades of the Science Complex and 2 façades of the Annex, at the Pacific Wildlife Research Centre, Delta, BC, Canada. [file peerj-10-13142-s001.docx]

|  | Fall | | Spring | Summer | Winter | Total No. Surveys |
| --- | --- | --- | --- | --- | --- | --- |
| Conventional glass (Science Complex) and ORNILUX glass (Annex) | | | | | | |
| 2013 | 51 | | 37 | 54 | 14 | **156** |
| 2014 | 39 | | 43 | 45 | 43 | **170** |
| 2015 |  | | 13 |  | 23 | **36** |
| Total surveys  in 730 day period | 90 | | 93 | 99 | 80 | **362** |
| Feather Friendly^®^ glass (Science Complex) and ORNILUX glass (Annex) | | | | | | |
| 2016 | 36 | |  |  | 13 | **49** |
| 2017 | 31 | | 40 | 50 | 49 | **170** |
| 2018 | 12 | | 47 | 56 | 30 | **145** |
| Total surveys  in 730 day period | 79 | | 87 | 106 | 92 | **364** |
| Total all years | | **169** | **180** | **205** | **172** | **726** |
