# Supplementary material for: Bird protection treatments reduce bird-window collision risk at low-rise buildings within a Pacific coastal protected area
Source: PeerJ. 2022 Mar 22;10:e13142. doi: 10.7717/peerj.13142 (PMC8953498; doi:10.7717/peerj.13142)
Supplement: Supplemental Information 2 [file peerj-10-13142-s002.docx]

| Time Period | Conventional glass  (2013 - 2015) | Feather Friendly^®^  (2016 - 2018) | ORNILUX  (2013 - 2015) | ORNILUX  (2016 - 2018) | |
| --- | --- | --- | --- | --- | --- |
| 1. Standardized Surveys | | | | | |
| Collisions detected | 33 | 2 | 3 | 4 |  |
| Species | Golden-crowned Kinglet (1)  Warbling Vireo (1)  Barn Swallow (1)  Hermit Thrush (2)  Dark-eyed Junco (2)  Song Sparrow (1)  Golden-crowned Sparrow (1)  Fox Sparrow (1)  Unknown (23) | European Starling (1)  Unknown (1) | House Finch (1)  Unknown (2) | Belted Kingfisher (1) Unknown (3) | |
| 1. Incidental Observations | | | | | |
| Collisions detected | 16 | 2 | 5 | 6 | |
| Species | Black-capped Chickadee (2)  Golden-Crowned Kinglet (2)  Barn Swallow (2)  American Robin (1)  Spotted Towhee (2)  Sparrow spp. (4)  Unknown (3) | Fox Sparrow (1)  Varied Thrush(1) | Barn Swallow (1)  Song Sparrow (1)  Orange-crowned Warbler (1)  Unknown (5) | Hermit Thrush (2)  American Robin (1)  Fox Sparrow (3)  Lincoln’s Sparrow (1) | |
| TOTAL  Collisions detected | 49 | 4 | 8 | 9 | |
|  | unknown fate (16)  carcasses (33) | unknown fate (1)  carcasses (3) | unknown fate (7)  carcasses (1) | unknown fate (3)  carcasses (6) | |
