# Supplementary material for: Bird protection treatments reduce bird-window collision risk at low-rise buildings within a Pacific coastal protected area
Source: PeerJ. 2022 Mar 22;10:e13142. doi: 10.7717/peerj.13142 (PMC8953498; doi:10.7717/peerj.13142)
Supplement: Supplemental Information 3 [file peerj-10-13142-s003.docx]

|  | **Science Complex**  **Feather Friendly^®^ glass** | | **Annex**  **Ornilux** | |  |
| --- | --- | --- | --- | --- | --- |
|  | **2013-2015** | **2016-2018** | **2013-2015** | **2016-2018** | **Total** |
| **Fall** | 11 | 0 | 1 | 1 | 13 |
| **Spring** | 7 | 0 | 0 | 0 | 7 |
| **Summer** | 3 | 0 | 1 | 1 | 5 |
| **Winter** | 12 | 2 | 1 | 2 | 17 |
| **Grand Total** | 33 | 2 | 3 | 4 | 42 |
